# On-chip coherent microwave-to-optical transduction mediated by ytterbium in YVO_4_

**DOI:** 10.1038/s41467-020-16996-x

**Published:** 2020-06-29

**Authors:** John G. Bartholomew, Jake Rochman, Tian Xie, Jonathan M. Kindem, Andrei Ruskuc, Ioana Craiciu, Mi Lei, Andrei Faraon

**Affiliations:** 1 Thomas J. Watson, Sr., Laboratory of Applied Physics, California Institute of Technology, Pasadena, CA 91125 USA; 2 Kavli Nanoscience Institute, California Institute of Technology, Pasadena, CA 91125 USA; 3 Institute for Quantum Information and Matter, California Institute of Technology, Pasadena, CA 91125 USA; 4 Present Address: School of Physics, The University of Sydney, Sydney, NSW 2006 Australia; 5 Present Address: University of Sydney Nano Institute, University of Sydney, Sydney, NSW 2006 Australia; 6 Present Address: JILA, University of Colorado and NIST, Boulder, CO USA; 7 Present Address: Department of Physics, University of Colorado, Boulder, CO USA; 8 Present Address: National Institute of Standards and Technology (NIST), Boulder, CO USA

**Keywords:** Quantum information, Nanophotonics and plasmonics

## Abstract

Optical networks that distribute entanglement among various quantum systems will form a powerful framework for quantum science but are yet to interface with leading quantum hardware such as superconducting qubits. Consequently, these systems remain isolated because microwave links at room temperature are noisy and lossy. Building long distance connectivity requires interfaces that map quantum information between microwave and optical fields. While preliminary microwave-to-optical transducers have been realized, developing efficient, low-noise devices that match superconducting qubit frequencies (gigahertz) and bandwidths (10 kilohertz – 1 megahertz) remains a challenge. Here we demonstrate a proof-of-concept on-chip transducer using trivalent ytterbium-171 ions in yttrium orthovanadate coupled to a nanophotonic waveguide and a microwave transmission line. The device′s miniaturization, material, and zero-magnetic-field operation are important advances for rare-earth ion magneto-optical devices. Further integration with high quality factor microwave and optical resonators will enable efficient transduction and create opportunities toward multi-platform quantum networks.

## Abstract (web summary)

Long distance interfaces between superconducting quantum information processing nodes would require coherent, efficient and low-noise microwave-to-optical conversion. Here, the authors use Yb ion ensembles in yttrium orthovanadate to demonstrate a transducer with the potential to fulfill these requirements.

## Introduction

Rare-earth ion (REI) ensembles simultaneously coupled to optical and microwave resonators have been proposed for microwave-to-optical (M2O) transducers^1,2^ that could achieve an efficiency and bandwidth to challenge other leading protocols^3,4^. A further advantage of the REI platform compared to electro-optical^5,6^, electro-optomechanical^7,8^, piezo-optomechanical^9,10^, and other magneto-optical^11^ schemes is the existing REI infrastructure for building complex quantum-optical networks^12^ including sources^13–15^ and memories^16–18^ for quantum states of light. While REIs provide promise for future networks, transducer demonstrations have been limited to macroscopic devices^19,20^. These millimeter-scale transducers currently require high optical pump powers that will be challenging to integrate with cryogenic cooling systems and light-sensitive superconducting circuits^19^. In contrast, on-chip REI technologies provide strong optical mode confinement to reduce the required pump power by several orders of magnitude, and miniaturization expedites integration of multiple devices for powerful control of photons at the quantum level. To achieve further integration with superconducting qubit platforms, it is also highly beneficial to extend REI schemes^1,2^ to zero magnetic field operation^21^. Toward this end, trivalent ytterbium-171 (^171^Yb^3+^) is appealing because it exhibits the simplest spin-state structure with gigahertz-frequency hyperfine transitions^22,23^.

**Fig. 1:**
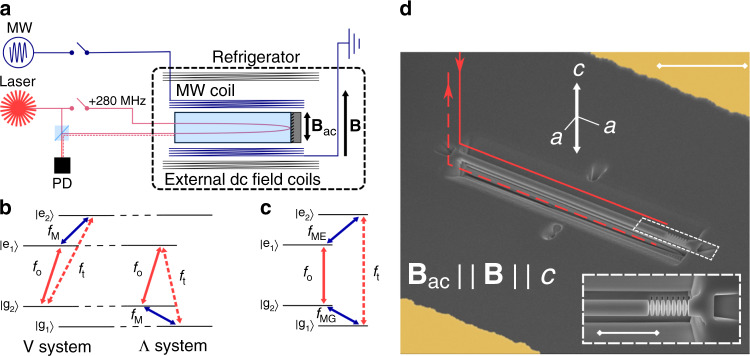
Concept and miniaturized implementation of a rare-earth ion magneto-optic modulator. **a** Conceptual schematic of the REI magneto-optic modulator. A microwave field **B**_ac_ is transduced to an optical field (dotted coral line) using a REI ensemble in a crystal. The crystal is coupled to a microwave transmission line (MW coil) and pumped by a laser field (solid coral line). Magnetic field coils provide control of the external dc field **B**. The transduced signal is combined with a frequency-shifted local oscillator on a photodiode to provide high signal-to-noise ratio heterodyne detection. **b** Example three-level energy structures proposed for REI magneto-optic transducers with the input microwave (*f*_M_), optical pump (*f*_o_), and transduced optical output (*f*_t_). **c** Example four-level energy structure for transduction in zero magnetic field with an additional microwave pump (*f*_MG_). **d** False color scanning electron microscope image of the planar, on-chip realization of the device in panel **a** (length of scale bar is equivalent to 10 μm). The 30 μm-long waveguide had a single photonic crystal mirror defined for the transverse magnetic mode (see inset: length of scale bar is equivalent to 4 μm). Light was coupled to and collected from the device using the coupler formed from a 45° cut at one end of the waveguide (indicated by coral lines). The gold coplanar waveguide provided a microwave frequency oscillating magnetic field aligned with the crystal *c*-axis, while a home-built superconducting solenoid (not shown) provided an external dc field, also aligned with the crystal *c*-axis.

We report a miniaturized magneto-optic modulator based on ^171^Yb^3+^-doped yttrium orthovanadate (YVO_4_) that allowed low-efficiency coherent M2O transduction at near-zero and zero magnetic field. The concept for the proof-of-principle device is shown in Fig. 1a–c. The REI crystal was cooled and simultaneously coupled to optical and microwave excitations. The coherence generated on the spin transition from excitation at frequency *f*_M_ (3.4 GHz) is mapped to an optical coherence at frequency *f*_t_ (304,505 GHz) through an optical pump field at frequency *f*_o_. We measured the transduced signal at *f*_t_ using optical heterodyne detection with a strong local oscillator at frequency (*f*_o_—280 MHz). The efficiency of the transduction is currently limited by the weak coupling between the ion transitions and the optical and microwave modes in the device′s broadband waveguides. Future devices will harness on-chip cavities to increase the mode coupling to progress toward efficient transduction operating at a quantum level.

## Results

### ^171^Yb^3+^:YVO_4_ device concept and fabrication

A 30 µm-long nanophotonic waveguide was fabricated in one of the gaps between the ground and signal lines of a gold microwave coplanar waveguide (Fig. 1d). A photonic crystal mirror fabricated on one end of the waveguide allowed optical fields to be launched and collected from a single 45° coupler on the opposite end of the device. We used waveguides to enable operation over a wide frequency range and to test multiple optical and microwave transitions but using cavities rather than waveguides in future devices will dramatically increase the efficiency of the transduction process^19,20^. The device was thermally contacted to a dilution refrigerator with a base temperature of ~30 mK (see Methods section, and Supplementary Note 1 for further details). While the device thermalized to 40 ± 10 mK, the waveguide temperature during continuous transduction was estimated to be ~1 K due to the heating of the optical pump (see Supplementary Note 9 for details).

To achieve efficient M2O transduction using REI-doped crystals, it is critical to have an ensemble with low inhomogeneity and cavity coupled optical and microwave transitions with collective cooperativities greater than unity^1^. The properties of ^171^Yb^3+^:YVO_4_ can satisfy both requirements^22^. Significantly, the ^171^Yb^3+^ optical transition near 984.5 nm exhibits a narrow inhomogeneous linewidth (*Γ*_ih,o_ ≈ 200 MHz at a doping concentration of ~100 ppm), and a large 4*f*–4*f* oscillator strength (*f* = 5.3 × 10^−6^), resulting in a magneto-optic nonlinear coefficient 100× larger than other REI-doped crystals considered for transduction (see Supplementary Table 1 for details and Supplementary Note 2 for an overview of ^171^Yb^3+^:YVO_4_ energy levels).

### ^171^Yb^3+^ ion optical properties and transduction strategies

**Fig. 2:**
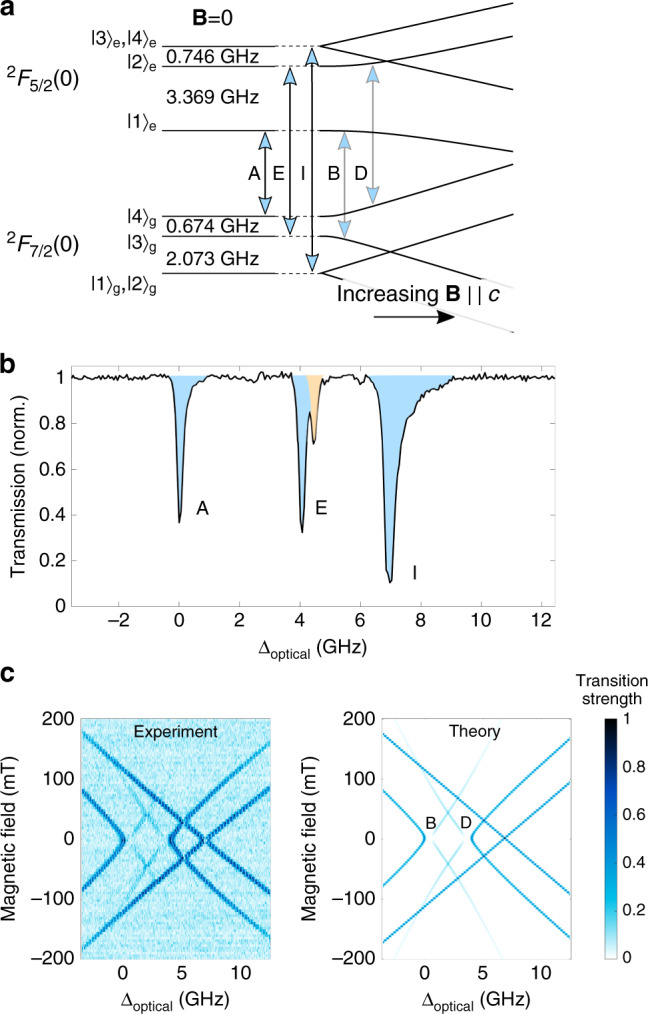
Magnetic field dependence of ^171^Yb^3+^:YVO_4_ optical transition frequencies and strengths. **a** Energy level structure for ^171^Yb^3+^:YVO_4_ with the permitted optical transitions for light polarized along the *c-*axis. Transitions A (304,501.0 GHz ≈ 984.54 nm), E, and I are the allowed, spin-preserving transitions at zero magnetic field, whereas transitions B and D only become allowed for |**B**| ≠ 0. **b** Transmission spectrum of the Yb^3+^:YVO_4_ nanophotonic waveguide (total length ≈ 60 µm) at a temperature of ~1 K at |**B**| = 0. The light is polarized along the *c*-axis and the spectrum is normalized to the transmission far off-resonance. The ^171^Yb^3+^ transitions (A, E, and I) are shaded blue and the impurity ^even^Yb^3+^ transition is shaded orange. **c** Comparison of the magnetic field-dependent relative transition strengths of ions in the waveguide device compared to the predicted transition strengths from spin Hamiltonian theory^22^. Each horizontal slice of the two-dimensional Experiment data is a normalized transmission spectrum like that in **b**. The level of absorption is proportional to the transition strength.

Figure 2a illustrates the zero-field energy levels of ^171^Yb^3+^ in YVO_4_. For light polarized parallel to the crystal *c*-axis, only the spin preserving transitions (A, E, and I) are allowed. The relatively large hyperfine interaction means that the three optical transitions are easily resolved in a waveguide transmission spectrum at zero magnetic field (Fig. 2b). Figure 2 highlights that there are no V- or Λ-systems available for transduction with magnetic field magnitude |**B** | = 0 for this polarization (the statement also holds true for the orthogonal polarization, as demonstrated in Supplementary Notes 7 and 8). We pursued two strategies to mediate transduction. First, we created suitable three-level systems by applying small magnetic fields along the *c*-axis, which introduced spin-state mixing through the linear Zeeman interaction. Second, we demonstrated a four-level scheme that overcomes the need for applied magnetic fields. In both cases we transduced microwave photons coupled to the spin transition in the optical-excited state, which will allow future transducers to benefit from decreased parasitic loss and dephasing due to coupling with spectator-ion ensembles^24^.

Figure 2c shows the normalized optical absorption of the ions in the waveguide as a function of magnetic field compared to the predictions of the ^171^Yb^3+^ spin Hamiltonian model^22^. Transitions B and D become allowed for non-zero magnetic fields, which can be used to form two V-systems and two Λ-systems. We transduced classical microwave signals using the V-systems containing the |1〉_*e*_ ↔ |2〉_*e*_ transition: *f*_M_ = 3.369 GHz at |**B**| ≈ 0 (Fig. 2a).

### M2O transduction using the V-system in a non-zero magnetic field

**Fig. 3:**
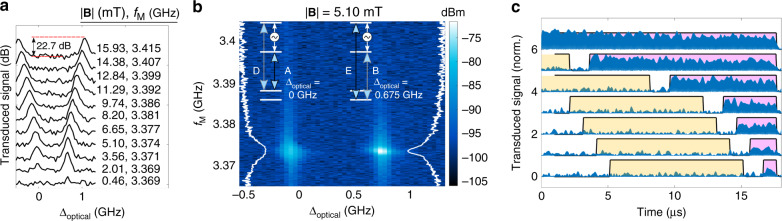
Continuous wave and pulsed microwave-to-optical transduction from the waveguide device. **a** Transduction signal produced at the D and E optical transition frequencies as a function of the applied field along the *c*-axis. The transduction is mediated by optically driving transitions A (*∆*_optical_ = 0 GHz) and B (*∆*_optical_ = 0.675 GHz) in their respective V-systems. The signal is optimized when the input microwave field frequency *f*_M_ is resonant with the excited state hyperfine transition at ~3.4 GHz. **b** A double resonance scan showing the transduced signal as a function of both the optical and microwave frequencies, which provides an indication of the inhomogeneous broadening of the relevant transitions. (Detection bandwidth = 3 kHz, optical pump power in the waveguide = 2 µW, Rabi frequency *Ω*_o_ ≈ 6 MHz, and microwave power of −5.3 dBm in the coplanar waveguide, Rabi frequency *Ω*_m_ ≈ 1 MHz.) White curves show the transduced signal (log scale) as a function of *f*_M_ at the middle of the optical inhomogeneous line. **c** Pulsed transduction signals (offset for clarity) generated at *f*_t_ (blue) at the maximum efficiency point in **b**. The yellow pulse indicates excitation at *f*_o_ only, whereas during the purple pulses the ensemble is excited with both *f*_o_ and *f*_M_ generating the transduced field.

Figure 3a shows example M2O transduction signals using the three-level strategy as a function of laser excitation frequency for increasing magnetic field. When |**B**| ≠ 0 and the ions are optically driven on transition A (B) at an offset frequency *∆*_optical_ around 0 GHz (0.675 GHz), microwave tones resonant with the excited state transition are transduced to optical photons emitted on the D (E) transition. Without cavity enhancement the transduction signal is strongest for input fields resonant with the ion transitions, whereas cavity coupling would allow high efficiency off resonance^1,25^. As the magnetic field increases, the transduced signal magnitude varies as the dipole moments and inhomogeneity of the optical and spin transitions change. Figure 3b shows a double resonance scan showing the transduced signal intensity as a function of the pump frequency and the applied microwave frequency for |**B**| = 5.1 mT (see Supplementary Notes 7 and 8 for additional data).

The high signal-to-noise ratio data was enabled by the optical heterodyne detection, which overcomes the low device photon-number efficiency *η* = 1.2 × 10^−13^ (see Supplementary Note 3). Given the characterization of our material, temperature, and driving rates we expect to increase the device efficiency by a factor ≥3 × 10^12^ by targeting optimized microwave and optical cavity coupling (see Supplementary Note 4), and applying the optical pump within the same cavity mode resonant with the signal. That is, the same ensemble of ^171^Yb^3+^ ions coupled to one-sided microwave and optical resonators, each with a quality factor of 2 × 10^4^, could perform at an overall *η* > 0.3 with improvements to the optical coupling efficiency into a single mode fiber (see Supplementary Note 4). The dramatic increase in efficiency is possible because *η* scales quadratically with the photon–ion coupling strength for *η* ≪ 1^19,20^.

To characterize the transducer′s bandwidth, we performed pulsed M2O transduction measurements (shown in Fig. 3c). The decrease in signal for pulse lengths <10 µs suggests a bandwidth limited by the spin transition inhomogeneity $\varGamma _{{\mathrm{ih}},\,{\mathrm{s}}} \approx 100$ kHz, which was confirmed by ensemble Rabi flopping measurements ($\varGamma _{{\mathrm{ih}},\,{\mathrm{s}}} = 130$ kHz—Supplementary Note 5). The current bandwidth is similar to leading electro-optomechanical^8^ transducers but lower than the megahertz-bandwidths demonstrated in other schemes^4^ including REI demonstrations^19,20^. The bandwidth could be increased by intentionally broadening $\varGamma _{{\mathrm{ih}},\,{\mathrm{s}}}$ through increased dopant concentration or strain.

Performing transduction in atomic systems enables quantum memories to be incorporated directly into the transduction protocol^2^ to enable synchronization of network links. The coherence lifetime of the spin transition *T*_2 (Spin)_ sets an upper bound on the potential storage time. Using two-pulse Hahn echoes we measure *T*_2 (Spin)_ = 35 µs as |**B**| → 0 (see Supplementary Note 5), which is sufficiently long to enable useful storage relative to the timescales of typical microwave qubit operations (10–100 ns).

### M2O transduction using a four-level system for |B| = 0

**Fig. 4:**
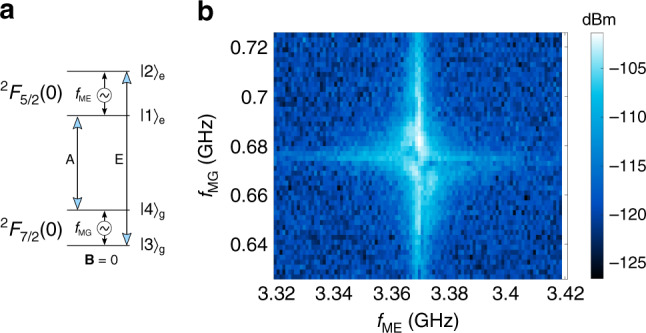
Four-level system transduction at zero field. **a** The energy levels used for a four-level magneto-optical transduction scheme at zero magnetic field using ^171^Yb^3+^:YVO_4_. The input microwave field at the excited state hyperfine transition frequency *f*_ME_ is transduced to an output optical field resonant with transition E. The ions are pumped by a microwave field resonant with the ground state hyperfine transition *f*_MG_ and an optical field resonant with transition A. **b** Transduced signal at the frequency of the optical transition E as a function of the two microwave input signals with the detuning of the optical pump *∆*_optical_ = 0, which provides an indication of the inhomogeneous broadening of the relevant transitions (detection bandwidth = 30 Hz, optical pump power in the waveguide = 25 µW, Rabi frequency *Ω*_o_ ≈ 20 MHz, and microwave power of 3.7 dBm in the coplanar waveguide, Rabi frequency *Ω*_ME_ ≈ 3 MHz, *Ω*_MG_ ≈ 10 MHz).

Using a coherent three-level atomic system is a conceptually simple route toward transduction between the microwave and optical domains. There are, however, disadvantages to this scheme. Given a fixed pump field, the strength of the optical photon–ion coupling is reduced by at least a factor of 4 when using a V-system or Λ-system. This is because the total oscillator strength of the optical transition must be divided between the two optical branches. Also, operating with a small bias magnetic field is not ideal as it will require shielding for integration with superconducting qubits. We present an alternate transduction strategy using a four-level system driven by an optical and a microwave pump as shown in Fig. 4a. The ideal implementation of this method harnesses the full optical oscillator strength of the ions and for ^171^Yb^3+^:YVO_4_ the four-level scheme enables transduction at zero magnetic field. The tradeoff for moving to the four-level scheme is the need for an additional microwave drive tone resulting in more stringent device criteria to operate at high efficiency (see Supplementary Note 6).

Figure 4b shows a double resonance spectrum for the two microwave inputs, with the optical pump field fixed at the frequency of maximum transduction (*∆*_optical_ = 0). In our waveguide device the four-level scheme is less efficient than the three-level scheme and thus, requires increased laser power to measure the signal. The resultant increase in device temperature broadens the spin inhomogeneous linewidth, which in turn decreases the efficiency further. The signal modulation near resonance for both microwave fields is most likely produced by coherent destructive interference at specific population differences between the four levels^25^.

## Discussion

This waveguide device illustrates the appeal of miniaturized REI devices for quantum photonic applications. We have demonstrated coherent M2O transduction, presented a strategy to improve the efficiency to >30%, and extended the protocol to zero-magnetic-field operation. The enabling high spectral density of the ^171^Yb^3+^ transitions can also be applied to realize other quantum photonic interfaces such as sources and memories (see Supplementary Note 10 for preliminary optical measurements). Future work will target highly efficient transducers that will allow a detailed noise analysis of the protocol, and ultimately their integration with photonic quantum memories^23^ and ^171^Yb^3+^-ion single photon sources^15^ to create the interfaces for hybrid quantum networks.

## Methods

### Device

A 5 nm-thick layer of chromium and a 115 nm-thick layer of gold was deposited on a 3 × 3 × 0.5 mm (*a* × *a* × *c*) 86 ppm ^171^Yb^3+^-doped YVO_4_ crystal (Gamdan Optics) using electron beam evaporation (CHA Industries Mark 40). A coplanar waveguide was fabricated from the gold layer using electron beam lithography (Raith EBPG 5000+) followed by wet-etching in gold etchant.

The photonic structures were milled within the coplanar waveguide gaps using a Ga^+^ focused ion beam (FEI Nova 600 Nanolab). The underlying structure for the nanophotonic waveguide is a suspended beam with an equilateral triangular cross section with each side equal to ~1 µm. A distributed Bragg reflecting mirror was then milled into the waveguide, using similar cuts used to define photonic crystal resonators in our previous work^26^.

### Experimental setup

The device chip was bonded to an oxygen-free high thermal conductivity (OFHC) copper sample holder using a thin layer of silver paint (Pelco). The gold coplanar waveguide was wire bonded to a PCB board from Montana Instruments fitted with SMP-type coaxial connectors. The sample holder was incorporated into a home-built, OFHC copper apparatus attached to the mixing chamber of a BlueFors dilution refrigerator. The apparatus incorporates a homebuilt superconducting solenoid (field coefficient = 77.3 mT/A) and a fiber-coupled-lens pair mounted onto a three-axis nanopositioner (Attocube).

Continuous-wave transduction measurements were made using a Field Fox N9115A spectrum analyzer. Optical signals from the device were combined with a strong optical local oscillator on a 50:50 fiber beam splitter. The output from the beam splitter was detected by an InGaAs fiber-coupled photodetector with a 5 GHz bandwidth (Thorlabs DET08CFC). The output from the detector was filtered using a bias-tee (Minicircuits ZFBT4B2GW+) and the strong beat signal at the local oscillator offset frequency (280 MHz) was suppressed using a band-block filter (RF Bay BSF-280M). The signal was then amplified (Pasternack PE15A1010) before being detected by the Field Fox receiver.

For the time domain measurements, the amplified signal was further amplified by two Minicircuits ZX60-3800LN-S+ amplifiers and mixed down (Minicircuits ZX05-30W-S+) to a frequency of 21.4 MHz using a local oscillator signal at ~3.6704 GHz (TPI-1002-A). The lower frequency signal was then filtered (Minicircuits BBP-21.4), amplified (SR445), and detected on a TDS7104 oscilloscope. To gate the microwave input to the device we used a Minicircuits ZASWA-2-50DR+ TTL-controlled switch.

The optical excitation was provided by a cw titanium sapphire laser (either M^2^ SolsTiS or Coherent MBR). For higher precision measurements, the SolsTiS was locked to an ultra-low expansion reference cavity (Stable Laser systems) with a controllable offset frequency provided by an electro-optic modulator (IX Blue). The laser light was fiber coupled and sent through a free space polarization controller. The polarized light was then split into two paths, one acting as the sample pump beam, and the other as the optical local oscillator. The pump beam was frequency shifted and gated through a fiber acousto-optic modulator (AOM—Brimrose) and input into the fridge using a circulator.

Absorption measurements were performed using a home-built external cavity diode laser. In this case, the transmitted light was detected by a switchable gain InGaAs photodetector (Thorlabs PDA10) or a Perkin Elmer APD. In the case of photon counting experiments, time tagging was performed by Sensl or Picoquant data acquisition electronics.

For pulsed all-optical measurements, the input light was gated using two double-pass AOMs (Intraction) and the signal gated by a third single-pass AOM before detection on the APD.

For further details refer to Supplementary Note 1 and Supplementary Fig. 1.

## Supplementary information

Supplementary Information

## Data Availability

The data that support the findings of this study are available from the corresponding author upon reasonable request.
