# Supplementary material for: On-chip coherent microwave-to-optical transduction mediated by ytterbium in YVO4
Source: Nat Commun. 2020 Jun 29;11:3266. doi: 10.1038/s41467-020-16996-x (PMC7324619; doi:10.1038/s41467-020-16996-x)
Supplement: Supplementary file 1 — Supplementary Information [file 41467_2020_16996_MOESM1_ESM.pdf]

Supplementary Information:

On-chip coherent microwave-to-optical transduction mediated by ytterbium in YVO_4

Bartholomew et al.

Supplementary Note 1 - Experimental setup

Here we include a detailed schematic of the experiment (Supplementary Figure 1.a) and its different configurations, along with further information to supplement the Methods section.

The device chip was thermally lagged to an oxygen-free, high thermal conductivity (OFHC) copper sample mount (Supplementary Figure 1.b) using silver paint. The gold coplanar waveguide fabricated on the YVO crystal surface was wire bonded to the PCB board as shown in Supplementary Figure 1.c. SMP connectors were contacted to the underside of the PCB board, which allowed the coaxial cables shown in Supplementary Figure 1.a to connect to the device.

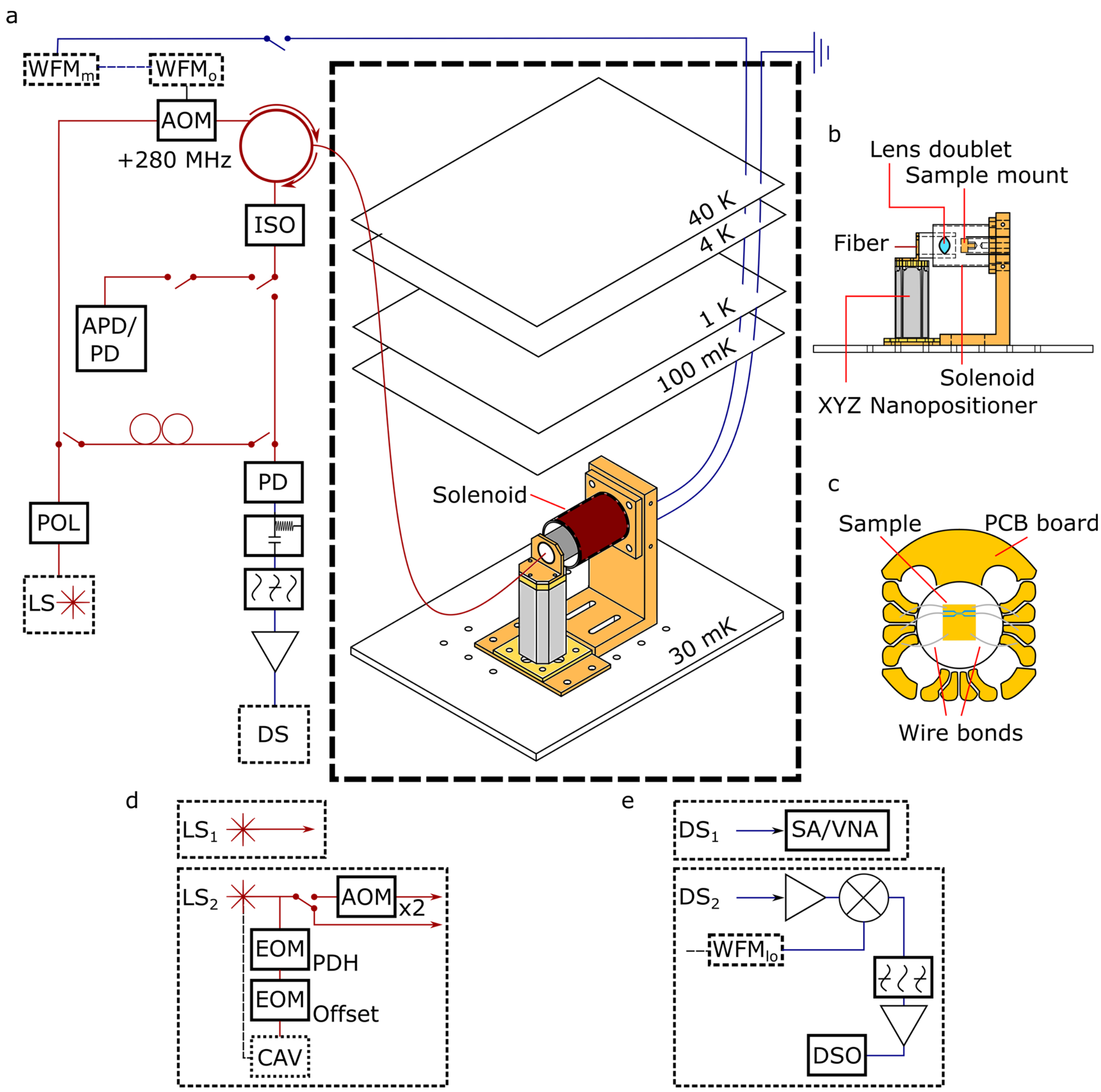

Supplementary Figure 1: Schematic of the experimental setup. a Apparatus for the experiments showing the optical path (red), the microwave path (blue), and the dilution refrigerator mounting. WFM_m - microwave waveform, WFM_o – optical waveform, AOM – acousto-optic modulator, ISO – fiber isolator, APD – avalanched photodiode, PD – photodiode, POL – polarisation control, LS – laser system, DS – detection system, SA – spectrum analyser, VNA – vector network analyser, EOM – electro-optic modulator, CAV – reference cavity, WFM_{lo} – local oscillator waveform, DSO – digital oscilloscope. **b** Cross-sectional view of the dilution refrigerator mounting. **c** View of the sample mount along the axis of the lens tube. **d** The two laser systems used in this work. **e** The two heterodyne detection systems used in this work.

Light from the laser system (LS) was coupled to the on-chip devices using a lens doublet mounted on an XYZ nanopositioner (Attocube). The excitation light was polarization controlled (POL), and was intensity modulated using a fiber acousto-optic modulator (AOM from Brimrose, centered at 280 MHz). Output light from the device was routed through a 90:10 fiber splitter, and a fiber isolator (ISO). For intensity detection, the light was routed to an AOM-gated avalanche photodiode (APD from Perkin Elmer) or InGaAs photodiode (PD from Thorlabs). For heterodyne detection, the light was routed to a high bandwidth photodiode after mixing with a strong local oscillator (LO) in a fiber beam splitter.

The electronic signal from the heterodyne PD was filtered using a bias-tee and a band-block filter (attenuating the strong signal at 280 MHz produced by the LO interfering with reflected pump light). The signal was then amplified and sent to one of the detection systems (DS) detailed in Supplementary Figure 1.e.

The optical waveform (WFM_o) was generated by an HP8656 B signal generator that was gated using a TTL controlled switch (Minicircuits ZASWA-2-50DR+) and amplified (Minicircuits ZHL-1-2W+). The microwave waveform (WFM_m) consisted of the amplified output signal from a spectrum analyser or vector network analyzer that was gated with a TTL switch. To maintain the fixed frequency and phase relationship of the electronic signals, all function generators were locked to the reference clock of the FieldFox N9115A. The total phase stability of the setup was limited to a few seconds because of temperature and position drift in the optical fibers.

LS_1 shown in Supplementary Figure 1.d used one of two lasers. A homebuilt external cavity diode laser (ECDL - built using the design outlined in [1]) was used for the inhomogeneous linescans. For transduction experiments we used a cw titanium-sapphire laser (Coherent MBR) locked to its own internal reference cavity. LS_2 used an M^2 SolsTiS offset-locked to an ultra-low expansion reference cavity using two electro-optic modulators. The light could be gated using two double-pass AOM setups or routed directly to the experiment.

DS_1 shown in Supplementary Figure 1.e was used for continuous wave transduction measurements. The detector was a FieldFox N9115A spectrum analyser (SA), or a Copper Mountain C1209 vector network analyzer (VNA) for phase sensitive measurements. DS_2 was used for pulsed transduction measurements. The electronic signal from Supplementary Figure 1.a was amplified, mixed down to 21.4 MHz using a local oscillator, filtered, and further amplified before detection on a digital oscilloscope.

Device details

The sample used in this work was cut from an yttrium orthovanadate boule doped with isotopically enriched (95%) $^{171}Yb^{3+}$ (Gamdan Optics). The $^{171}Yb^{3+}$ concentration was determined to be 86 ppm relative to the host yttrium using glow discharge mass spectrometry (GDMS - EAG Laboratories).

The 3 x 3 x 0.5mm (a x a x c) sample was cut and polished by Brand Optics. Following the chromium and gold deposition, a ZEP mask was defined by electron beam lithography (Raith EBPG 5000+). The samples were then wet-etched in gold etchant to form the coplanar waveguide. The 65 µm wide conductor was centered between the two ground planes with the edge-to-edge distance from conductor to ground plane approximately 50 µm. The resist was then removed with Remover PG.

A further 50 nm of chromium was then evaporated onto the sample as a hard mask. The sample was milled using a Ga+ focused ion beam (FEI Nova 600 Nanolab). The underlying structure for the nanophotonic waveguide was a suspended beam with an equilateral triangular cross section, with each side equal to approximately 1 µm. A distributed Bragg reflecting mirror was then milled into one end of the waveguide[2], along with the 45° couplers. The chromium layer was then removed using chrome etchant (CR-7).

Supplementary Note 2 - Crystal structure, site symmetry, and energy levels

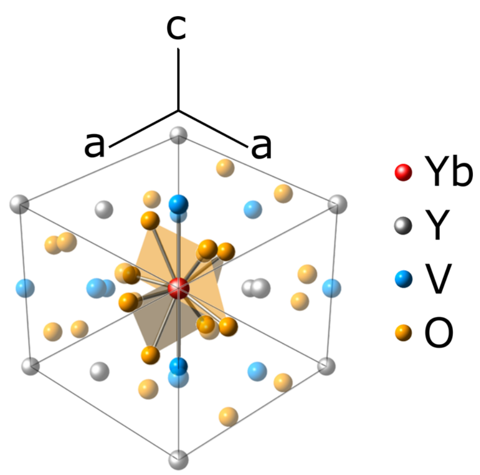

Supplementary Figure 2: Unit cell of YVO_4. An Yb^{3+}-ion has substituted for the central Y^{3+}-ion in the cell.

Yttrium orthovanadate (YVO) is a uniaxial crystal in which the Y^{3+}-ion sits at a site of D_{2d} symmetry. In Supplementary Figure 2 the two orthogonal trapezoids that connect the nearest 8 O^{2-}-ions give a guide to the eye for visualizing D_{2d} symmetry. Importantly, the space point group D_{2d} is non-polar, which means that substitutional Yb^{3+}-ions in this site have zero first order sensitivity to electric fields.

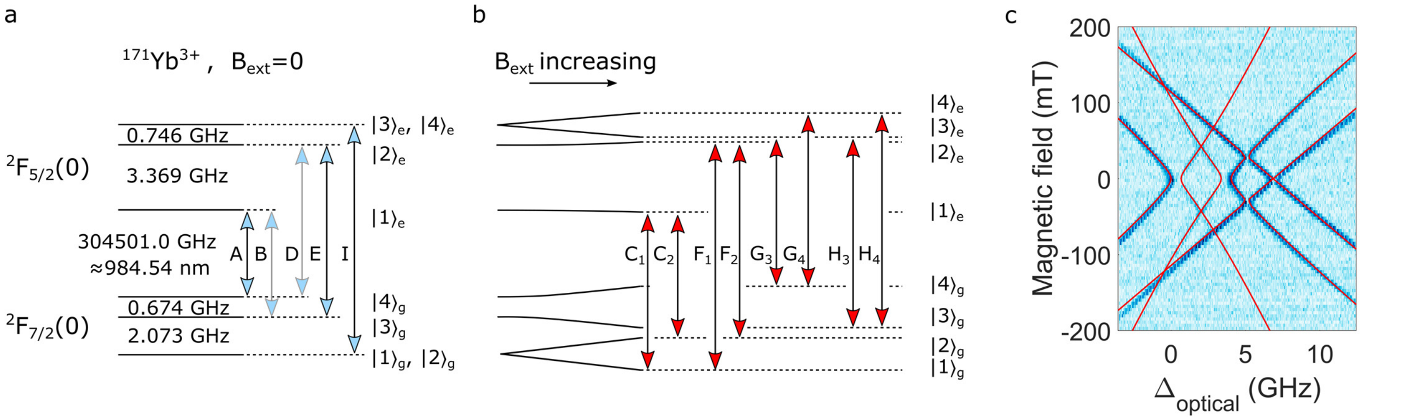

Supplementary Figure 3: Energy level diagrams of $^{171}Yb^{3+}$-ions in YVO. a Zero magnetic field energy levels and transitions polarized along the crystal *c* axis. **b** Non-zero field energy levels and transitions polarized perpendicular to the *c* axis. Transitions A, E, and I are allowed at zero field, as are transitions $C_{1,2}$, $F_{1,2}$, $G_{3,4}$, and $H_{3,4}$. **c** Repeated data from Figure 3(c) of the paper, where the predictions of the Hamiltonian model are superimposed (in red) on the experimental data.

Yb^{3+} has 13 electrons in the 4f shell, which yields a relatively simple electronic energy level structure (it is effectively a one-hole system). The degeneracy of the two spin-orbit manifolds $^2F_{7/2}$ (ground) and $^2F_{5/2}$ (excited) is lifted by the D_{2d}–symmetric crystal field interaction. We focus on the lowest lying levels of both multiplets, denoted in Supplementary Figure 3 as $^2F_{7/2}(0)$ and $^2F_{5/2}(0)$. Only $^2F_{7/2}(0)$ is thermally populated at liquid helium temperatures because it is separated from the next crystal field levels by >200 cm^{-1} (> 6 THz).

$^{171}Yb^{3+}$ has a nuclear spin of ½, which interacts with the ion electron spin to partially lift the remaining degeneracy at zero field. We transduce microwave photons using the $|1\rangle_e \leftrightarrow |2\rangle_e$ or $|3\rangle_g \leftrightarrow |4\rangle_g$ spin transitions, which have large transition strengths (the dipole moment is of the order of electron spins) for ac-magnetic fields applied along the crystal *c* axis. This is despite the states involved being hybridized electron-nuclear spin states. In a magnetic field, the remaining degeneracy is lifted, and transitions B and D become allowed because of the mixing between the hyperfine states.

Supplementary Note 3 - Device Efficiency measurement

To determine the device efficiency of the transducer we performed a calibration of the optical output losses, the microwave input losses, and the sensitivity of the heterodyne detection system.

The output efficiency with which a transduced photon from the waveguide reaches the photodiode was $\eta_{\mathrm{output}} = 0.09$. This encompasses the coupling efficiency between the free space lens doublet and waveguide ($\eta_{\mathrm{coupling}} = 0.22$) and losses in fiber connections, the optical isolator, and fiber beam splitters ($\eta_{\mathrm{optical\ path}} = 0.4$).

The microwave input coupling efficiency was dependent on frequency with $\eta_{\mathrm{input}}(3.369\ \mathrm{GHz}) = 0.15$ and $\eta_{\mathrm{input}}(0.674\ \mathrm{GHz}) = 0.45$. This was made up of the efficiency launching from coaxial cables into the waveguide { $\eta_{\mathrm{launch}}(3.369\ \mathrm{GHz}) = 0.74$, $\eta_{\mathrm{launch}}(0.674\ \mathrm{GHz}) = 0.88$ } and other system losses { $\eta_{\mathrm{mw\ path}}(3.369\ \mathrm{GHz}) = 0.20$, $\eta_{\mathrm{mw\ path}}(0.674\ \mathrm{GHz}) = 0.51$ }.

The heterodyne detection system was calibrated by measuring the beat note of two lasers (M^2 SolsTiS, and home built ECDL) locked at a frequency offset of 3.65 GHz. Using the measured detector responsivity (0.18 A/W) and the overall gain of the bias tee, filter, and amplifier (39.3 dB), the optical signal intensity producing the maximum electrical signal observed in the experiments -71.62 dBm (3 kHz BW) was calculated to be 280 fW . This corresponds to 1.4×10^6 photons/s at the output frequency.

Given the microwave input power of 3 dBm at a frequency of 3.369 GHz (8.9×10^{20} photons/s), and the efficiencies η_{output} and η_{input}, the photon number device efficiency of the transduction process $\eta = 1.2 \times 10^{-13}$.

Supplementary Note 4 - Increase in device efficiency by using cavities

In this section we detail the expected efficiency gains from several modifications to the dual waveguide device including the use of high quality-factor cavities rather than broadband waveguides. Using the model developed in Williamson *et al.* [3] the efficiency of the three-level transduction process where the ion ensemble is coupled to both a microwave and optical cavity is given by Supplementary Equation 1

$$\eta = \frac{4R^2}{(R^2+1)^2}\ \text{, for } R = \frac{2S}{\sqrt{\kappa_{\mathrm{o}}\kappa_{\mathrm{m}}}}\,. \tag{1}$$

The parameter S is the coupling strength between the microwave and optical cavities provided by the magneto-optic nonlinearity of the rare-earth ion ensemble. κ_{o} and κ_{m} are the decay rates of the optical and microwave cavities, respectively. R is the ratio of the coupling strength to the impedance-matched coupling strength, such that $\eta = 1$ when $R = 1$. R can be rewritten as[3]

$$R = \Omega\alpha F\sqrt{Q_{\mathrm{o}}Q_{\mathrm{m}}}\,, \tag{2}$$

where Ω is the Rabi frequency of the optical pump, α describes the density and spectroscopic properties of the ion ensemble (magneto-optical nonlinear coefficient), F is an effective filling factor describing the mode overlap of the three fields, and Q_{o} and Q_{m} are the quality factors of the one sided optical and microwave resonators, respectively. Although this theory is derived for the three fields being detuned from the relevant ion resonances (but in three photon resonance)[3], the formulation extends to the single pass regime[4]. Without cavities, the highest efficiency is achieved when the fields are resonant with the ion transitions[4].

For $R \leq 0.1$, η is approximately equal to $4R^2$: improvements to the current device coupling strength will contribute quadratically to the efficiency.

A significant gain in R can be made by using all the ions available. This increases α given that[3]

$$\alpha = \sqrt{\frac{\mu_0}{\hbar^2\epsilon_0}}\mu_{31}\mu_{21}\rho\int_{\epsilon_{\mathrm{m}}}^{\infty}\frac{D_{\mathrm{m}}(\delta_{\mathrm{m}})}{\delta_{\mathrm{m}}}d\delta_{\mathrm{m}}\int_{\epsilon_{\mathrm{o}}}^{\infty}\frac{D_{\mathrm{o}}(\delta_{\mathrm{o}})}{\delta_{\mathrm{o}}}d\delta_{\mathrm{o}}\ , \tag{3}$$

where ρ is the ion number density. The other parameters are denoted: μ_0 is the vacuum permeability, $\hbar$ is the reduced Planck constant, ϵ_0 is the vacuum permittivity, μ_{31} is the electric dipole moment for the output optical transition, μ_{21} is the magnetic dipole moment for the input microwave transition, and $D_\mathrm{m}(\delta_\mathrm{m})$ and $D_\mathrm{o}(\delta_\mathrm{o})$ are the functions describing the inhomogeneous broadening of the spin and optical transitions, respectively, which are assumed to be Gaussian.

For all the continuous wave transduction signals, the number of ions contributing to the signal was a factor of ≤0.25 of the available population. This is due to the thermal distribution of ions at the elevated temperatures (estimated to be approximately 1 K) caused by the continuous optical pump and input microwave field. Initializing the population into a non-thermal distribution through optical pumping will allow ρ to increase by a factor of 4. This is a conservative estimate for the current experiment given that optical pumping can contribute to population being trapped in spin states outside the V or Λ system.

The use of an optical cavity will reduce the power required to maintain the same optical pump Rabi frequency Ω, which will decrease the heat load on the device. If required, further decreases in device temperature can be achieved by running the transducer in a pulsed mode.

Replacing each waveguide with a cavity, will increase *R* by $\sqrt{\mathcal{F}_\mathrm{o}\mathcal{F}_\mathrm{m}}$, where $\mathcal{F}$ is the cavity finesse. Our group has already demonstrated the ability to fabricate photonic crystal mirrors to create cavities with $Q_\mathrm{o} > 2 \times 10^4$ [2]. For a 30 µm long optical cavity with a resonant frequency around 984.5 nm and $Q_\mathrm{o} = 10^4$, the cavity FWHM $\Delta f = 30.45$ GHz, and the finesse $\mathcal{F}_\mathrm{o} = 75.5$. Note that Δf is sufficiently large to allow the optical pump to be applied in the cavity mode resonant with the signal. This differs from the proposal of Williamson et al. [3] who apply the optical pump in a frequency-adjacent cavity mode (the optical FSR is matched with the input microwave frequency).

High quality factor microwave resonators can be fabricated on rare-earth ion host crystals using superconducting metals or alloys such as Nb or NbN [5,6]. For such cavities $\mathcal{F}_\mathrm{m} \approx Q_\mathrm{m}$, allowing $\mathcal{F}_\mathrm{m} \leq 2.5 \times 10^4$ without limiting the transduction bandwidth to less than the current inhomogeneity of the excited state transition (130 kHz).

For $Q_\mathrm{o} = Q_\mathrm{m} = 2 \times 10^4$, the predicted increase in *R* is approximately 1.7×10^3. Allowing for the more ambitious values $Q_\mathrm{o} = Q_\mathrm{m} = 10^5$, the feasible increase in *R* approaches 8.7×10^3, albeit accompanied by a reduction in the bandwidth to 34 kHz.

Another key factor in increasing *R* in the current transducer is increasing the effective filling factor F. Given the mode profiles for the two waveguides and the much larger size of the microwave waveguide compared to the optical waveguide, F can be approximated as

$$F \propto \sqrt{\frac{V_\mathrm{o}}{V_\mathrm{m}}}, \quad (4)$$

where V_o is the volume of the optical mode in YVO and V_m is the volume of the microwave mode on the chip. Several improvements can be made in F. First, the optical structure can be lengthened by a factor of 10 to 300 µm in length. Second, the coplanar microwave structure can be compacted by decreasing the conductor and gap widths by a factor of 20. A further order of magnitude improvement in F is possible by using a lumped element microwave cavity that concentrates the magnetic field in the mode volume of the optical cavity. This provides an increase in *R* by a factor of around 600.

We note that to maintain the adiabatic condition $\Omega^2 < \delta_\mathrm{o}\delta_\mathrm{m}$ [3] the current $\Omega \approx 6$ MHz would have to be decreased by approximately a factor of 2, reducing *R* by the same factor.

By optimizing α and F, and using microwave and optical cavity coupling the increase in R is predicted to reach a factor of 2×10^6. The corresponding increase in the device transduction efficiency according to Supplementary Equation 1 would yield $\eta \approx 0.4$. Such an efficiency is not fundamentally limited. Further increases to the cavity quality factors (to 10^5) and ^{171}Yb concentration are both feasible strategies for pushing toward unit efficiency.

The potential efficiency of a $^{171}Yb^{3+}$:YVO-based transducer (using the $|1\rangle_e \leftrightarrow |2\rangle_e$ transition) can also be considered by comparing its spectroscopic properties to Site 1 Er^{3+}:Y_2SiO_5 (Er:YSO), the material used in other transduction work[3,7,8].

**Parameter**	**^{171}Yb:YVO (86 ppm)**	**^{even}Er:YSO (10 ppm) (Site 1)**
$\rho_{\max}$ (assuming spin polarization)	1.08×10^{24} m^{-3}	9.35×10^{22} m^{-3}
Polarized optical oscillator strength f_{31}<br>Virtual cavity model<br>Real cavity model	 5.3×10^{-6} $(E \parallel c)$<br>1.4×10^{-5} $(E \parallel c)$	2×10^{-7} $(E \parallel D_2)$
Optical dipole moment μ_{31}<br>Virtual cavity model<br>Real cavity model	 6.4×10^{-32} C m<br>1.1×10^{-31} C m	2.13×10^{-32} C m
Spin dipole moment μ_{12}	17.6 GHz/T $(B \parallel c,\ B_{ac} \parallel c)$	35.5 GHz/T $(B \perp b$ & 29° to D_1, $B_{ac} \parallel c)$
$\Gamma_{\mathrm{ih\ (optical)}}$	200 MHz	500 MHz
$\Gamma_{\mathrm{ih\ (spin)}}$	0.13 MHz	1 MHz
α<br>(Calculated as detailed in [3])	1.6×10^{-8} s (Virtual cavity)<br>2.7×10^{-8} s (Real cavity)	4.8×10^{-11} s<br>$(1.4 \times 10^{-10}$ s for $\mu_{12} = 15\mu_{\mathrm{B}}/2$ as presented in [3])

Supplementary Table 1: Comparison of material spectroscopic properties. Note: calculating f_{31} and μ_{31} from the resonant absorption spectrum requires a local field correction[9,10]. Both the Virtual (or Lorentz) and Real (or Empty) cavity approximations are used in the literature and so we include both values here for ^{171}Yb:YVO based on our characterisation of this material[11].

As shown in Supplementary Table 1, ^{171}Yb:YVO has a value of α at least 100x greater than ^{even}Er:YSO. Therefore, for ^{171}Yb:YVO the value of $\Omega F\sqrt{Q_{\mathrm{o}}Q_{\mathrm{m}}}$ required for efficient transduction is correspondingly lower than for ^{even}Er:YSO. Our modelling of the filling factor F for on-chip transducer geometries suggests that F can approach the value achieved in the loop gap resonator geometry proposed in [3] and used in [7] ($F = 0.0084$). The work in [3] , predicts unit efficiency is possible for ^{even}Er:YSO with $Q_{\mathrm{o}}Q_{\mathrm{m}} = 10^{10}$. Based on Supplementary Table 1, efficient, on-chip ^{171}Yb:YVO transducers are feasible with cavity quality factors around 10^{4}. This correlates well with the analysis based on the current device.

All the analysis above considers a device efficiency with ideal one-sided cavities. The ultimate overall efficiency (output optical photons from a single mode fiber per input microwave photon) of the transducer will be dependent on $\kappa_{\mathrm{coupling}}/\kappa_{\mathrm{o}}$, where $\kappa_{\mathrm{coupling}}$ is the rate at which cavity photons are coupled into a single mode fiber. The best $\kappa_{\mathrm{coupling}}/\kappa_{\mathrm{o}}$ value for the current nanobeam devices[2,12] approach 0.15, but could be improved significantly by increasing device-fiber coupling with a higher NA coupling lens or moving to a hybrid platform that can enable higher efficiency photon extraction[13].

Supplementary Note 5 - Pulsed measurements of transducer bandwidth and coherence lifetime

Figure 3(c) of the main text shows pulsed transduction signals as a function of pulse length. In a pulsed regime the device temperature will be significantly lower than for the CW transduction studies (Supplementary Note 7), resulting in narrower spin inhomogeneous linewidth Γ_{ih} .

To measure Γ_{ih} we perform ensemble Rabi flopping using the pulse sequence shown in Supplementary Figure 4.a(i). The external field was 1.9 mT applied parallel to the *c* axis, with the laser excitation power = 2 μW in the waveguide and the microwave power = -5.3 dBm in the CPW. Population is initialised into $|1\rangle_e$ through the application of an optical pulse applied resonantly with transition B. The spin ensemble was then driven with a resonant microwave field near 3.369 GHz resulting in ensemble Rabi oscillations. In the regime where the microwave Rabi frequency is less than the optical pump Rabi frequency, the transduced field at frequency f_{t} is approximately proportional to the population difference between states $|2\rangle_e$ and $|1\rangle_e$. Thus, the amplitude of the transduced field produced by a combined optical-microwave readout pulse, measures the excited state spin inversion. The results are shown in Supplementary Figure 4.b. The damping rate of the Rabi oscillations due to the ensemble inhomogeneity is consistent with $\Gamma_{\mathrm{ih}} = 130$ kHz.

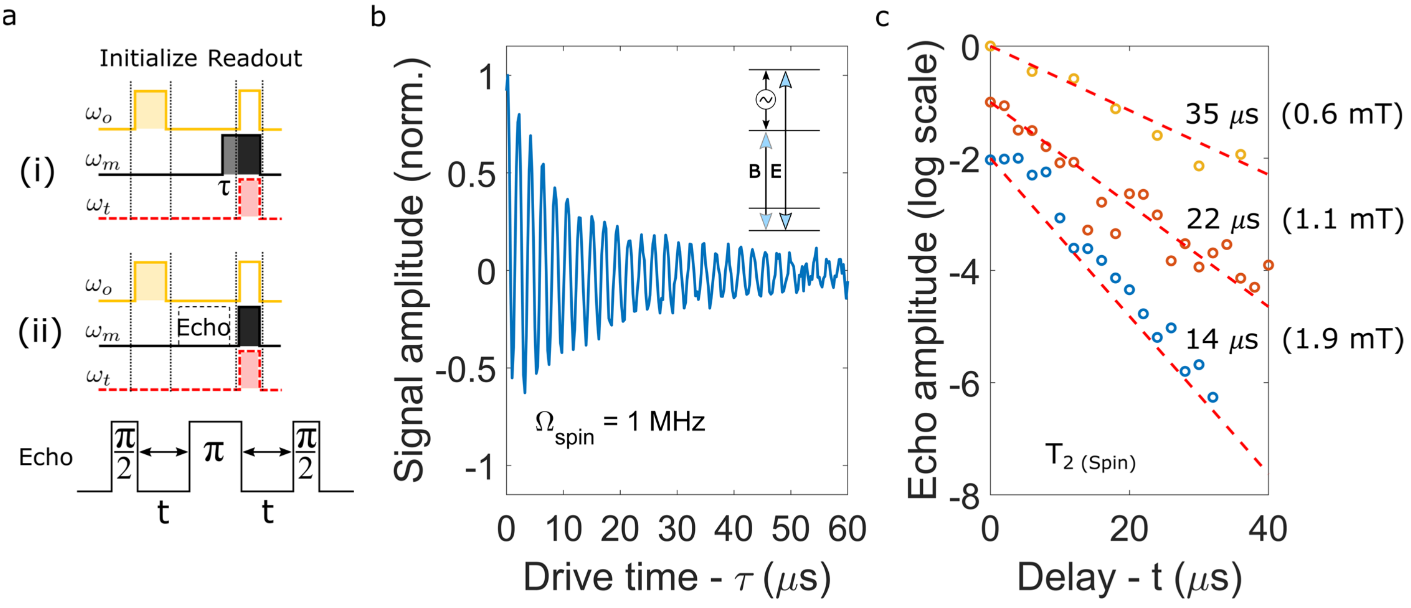

Supplementary Figure 4: Pulsed transduction measurements. a Pulse sequences for optical detection of excited state (i) spin-ensemble Rabi flopping, and (ii) spin transition Hahn echoes. **b** Excited state spin-ensemble Rabi flopping showing the narrow inhomogeneity of the transition and an effective spin Rabi frequency of 1 MHz. **c** Excited state spin transition Hahn echo decays as a function of sequence delay *t*. The coherence lifetime T_2 increases with decreasing field as the $^{171}Yb^{3+}$-ions approach a clock transition at **B** = 0.

We extended the pulse sequence as indicated in Supplementary Figure 4.a(ii) to measure the excited state spin coherence lifetime. In this case, a Hahn echo sequence was inserted between the initialization and readout steps of the sequence. The results are shown in Supplementary Figure 4.c. For an applied field of |**B**| = 1.9 mT parallel to the *c* axis the coherence lifetime was measured to be 14 μs. As the field was decreased to |**B**| = 1.1 mT (|**B**| = 0.6 mT) the coherence lifetime increased to 22 μs (35 μs). The increase in coherence as the $^{171}Yb^{3+}$ ions approach their zero-field clock transition indicates that the decoherence is dominated by magnetic noise from their environment. The experiments were limited to a minimum field of 0.6 mT because below this field the B transition becomes prohibitively weak and the transduction signal falls below our detection circuit noise.

Supplementary Note 6 - Four-level scheme

The four-level transduction scheme presented in the paper is analogous to the four-level transduction scheme proposed for cold gas atoms[14]. Therefore, the optical non-linearity, or coupling strength between the microwave and optical cavity is given by

$$S = \frac{\sqrt{N}\Omega_{12}\Omega_{23}g_{\mathrm{M}}\sqrt{N}g_{\mathrm{o}}}{\delta_2\delta_3\delta_4} F , \qquad (5)$$

where Ω_{12} is the microwave pump Rabi frequency on the $|3\rangle_g \leftrightarrow |4\rangle_g$ transition ($\mu = 42$ GHz/T), Ω_{23} is the optical pump Rabi frequency on the $|4\rangle_g \leftrightarrow |1\rangle_e$ transition (transition A), g_{M} is the single ion coherent coupling rate to the microwave field on transition $|1\rangle_e \leftrightarrow |2\rangle_e$ ($\mu = 17$ GHz/T), g_{o} is the single ion coherent coupling rate to the optical field on transition $|2\rangle_e \leftrightarrow |3\rangle_g$ (transition E), and the δ are the detunings relative to the upper three energy levels. We note that equivalently to the four-level scheme proposed in [14], there is no collective enhancement on the microwave transition targeted for transduction. Alternatively, a four-level scheme in $^{171}Yb^{3+}$:YVO could target the ground state spin transition for transduction, which would be collectively enhanced. The microwave pump would then be applied on the excited state spin transition.

The four-level scheme for transduction is advantageous in situations where the transducer is required to operate at zero field, or where the optical pump field needs to be minimized. In comparison to the three-level scheme[3,7,8], the four-level coupling strength S is modified by a factor of $\frac{\Omega_{12}}{\delta_2} < 1$ and a reduction due to the filling factor F that accounts for the overlap of a fourth field. Therefore, to achieve impedance matching for highly efficient transduction using the four-level scheme, the factor $\rho\sqrt{Q_o Q_{\mathrm{m}}}$ will have to increase to compensate, without adversely impacting other device parameters.

Supplementary Note 7 - Double resonance scans

Supplementary Figure 5 shows the transduction signal for the excited state microwave transition $|1\rangle_e \leftrightarrow |2\rangle_e$ with the optical pump polarization parallel to the crystal *c* axis. In part (a) the signal is shown as a function of the applied microwave frequency f_M, the optical pump frequency offset from transition A $\Delta_{\mathrm{optical}}$ and applied dc magnetic field along the crystal *c* axis. At zero field, there is no transduction signal because transitions B and D are forbidden (top left-hand plot). For non-zero fields, transduction is observed for two values of $\Delta_{\mathrm{optical}}$ corresponding to two V-systems: one containing transition A and the other containing transition B. At each magnetic field the signal is strongest when f_{M} and $\Delta_{\mathrm{optical}}$ correspond to center of the spin and optical inhomogeneous distributions, respectively.

In part (b) the overlay of 22 two-dimensional data sets illustrates the simultaneous evolution of the $|1\rangle_e \leftrightarrow |2\rangle_e$ microwave transition, and optical transitions A and B with the magnetic field. The spin Hamiltonian model developed in [11] accurately predicts the frequency evolution (red-dashed curves).

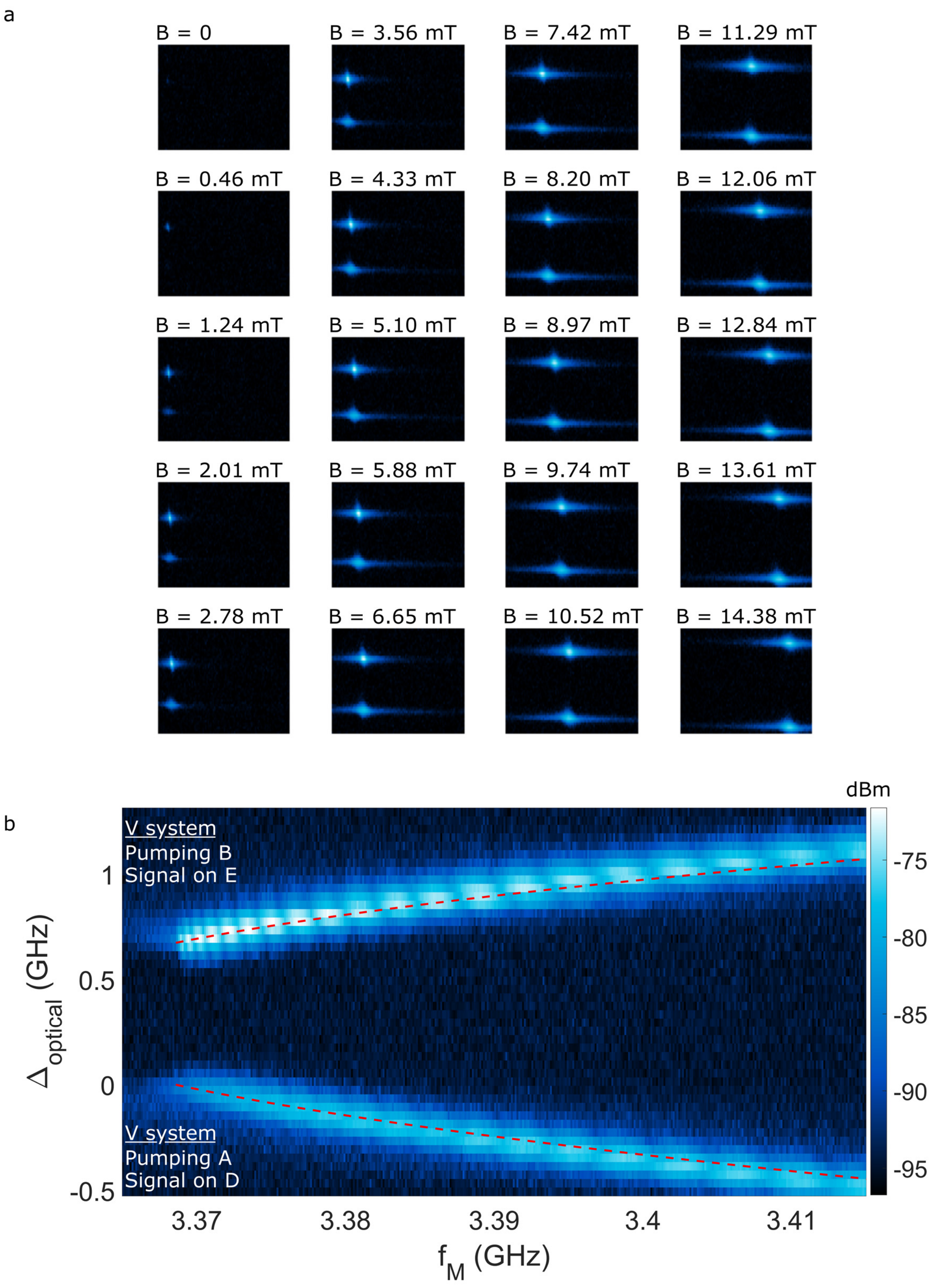

Supplementary Figure 5: The transduction signal as a function of microwave input frequency f_M and optical pump offset frequency $\Delta_{optical}$. a A series of 20 two-dimensional data sets showing the evolution of the transduced signal as the static magnetic field is increased along the crystal *c* axis. Note that the axes and color scaling matches the figure in part (b). **b** An overlay of 22 two-dimensional transduction data sets at varying fields. The red dashed curves are theoretical plots from the spin Hamiltonian model.

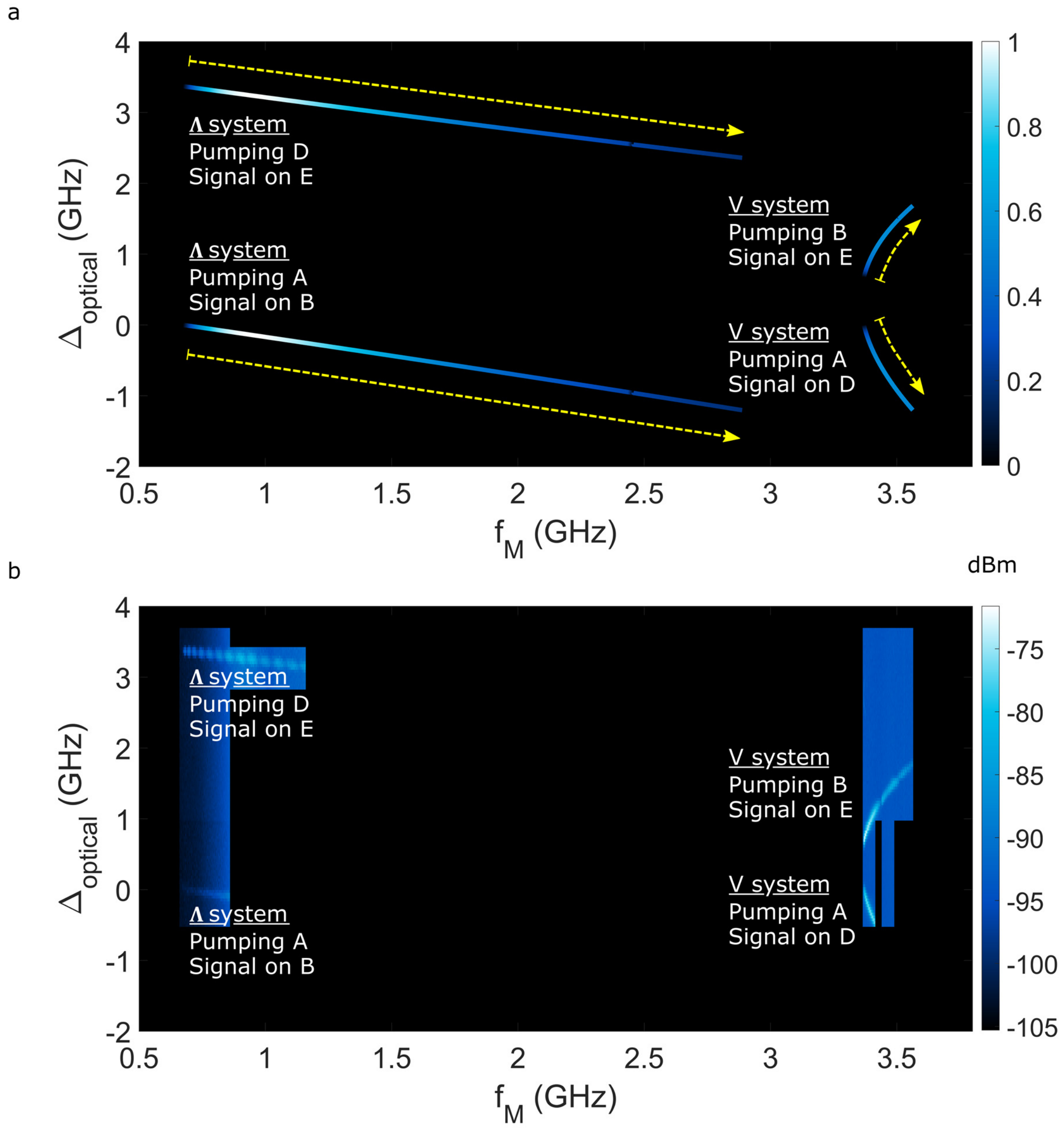

Supplementary Figure 6: Parameter space for transduction. a Predicted and, **b** observed parameter spaces for transduction given an increasing dc magnetic field, optical pump polarization, and microwave field all applied parallel to the crystal *c* axis. The dotted yellow arrows in (a) show the evolution with increasing field.

The signal strength is a function of several parameters that are magnetic field dependent. The spin Hamiltonian model can be used to predict the relative spin and optical dipole moments, which can be accurately approximated using the hyperfine and linear Zeeman parameters. Using this model the relative transduction signal strengths were calculated under the assumption of a fixed pump field. The modelling sampled magnetic fields $B \in [0, B_{max}] \,||\, c$ and the results are plotted for each of the two optical pump polarizations: $E \,||\, c$ in Supplementary Figure 6.a, and $E \perp c$ in Supplementary Figure 7.a. The maximum field is B_{max} = 33 mT and B_{max} = 38.7 mT in Supplementary Figures 6 and 7 respectively.

Supplementary Figures 6.b and 7.b show combined data sets from the on-chip waveguide. The measurements concentrate on a bandwidth of 200 MHz – 400 MHz in the microwave domain and 1-3 GHz in the optical domain close to the predicted signal maxima. In some cases this bandwidth was insufficient to capture the complete field dependent frequency evolution. In all cases the experimental data shows good agreement with the predicted parameter space for transduction.

a

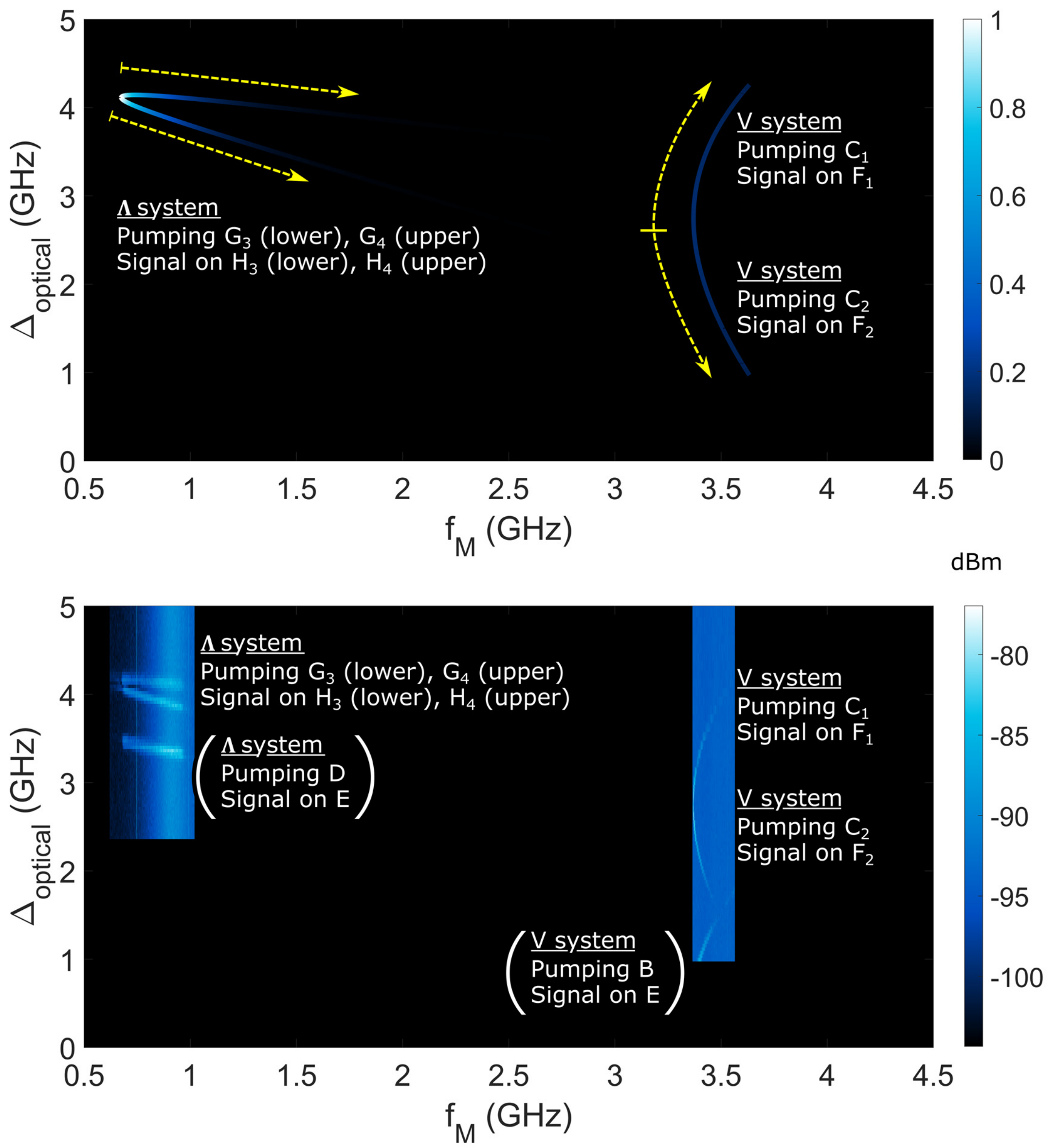

Supplementary Figure 7: Parameter space for transduction. a Predicted and, **b** observed parameter spaces for the signal given an increasing dc magnetic field, and microwave field applied parallel to the crystal *c* axis, and the optical pump polarization perpendicular to the *c* axis. The dotted yellow arrows in (a) show the evolution with increasing field.

There are several differences between the theoretical and experimental signal strengths in both Supplementary Figures 6 and 7. This is predominantly because the model does not account for the spin and optical transition inhomogeneous linewidths, which also vary with field. In Supplementary Figure 7, a further difference is observed. Both pairs of signals in Supplementary Figure 7.a are non-zero at zero applied field, whereas in the experimental data the transduction signal vanishes at zero field. As explored in Supplementary Note 8, at zero field there is destructive interference between two signals with opposite phase. This is not accounted for in the model, which calculates the relative magnitude rather than the amplitude of the transduced signal. Other differences are due to experimental artefacts. In Supplementary Figure 6.b the signal from the upper Λ transition appears in discrete lobes, which is due to coarse magnetic field steps. Similarly, the signal from the upper V system contains two discontinuities, which are due to polarization drift over the course of the data set. In Supplementary Figure 7.b the two signals specified in parentheses are from the residual light polarized along the *c* axis.

Supplementary Note 8 - Zero field cancellation

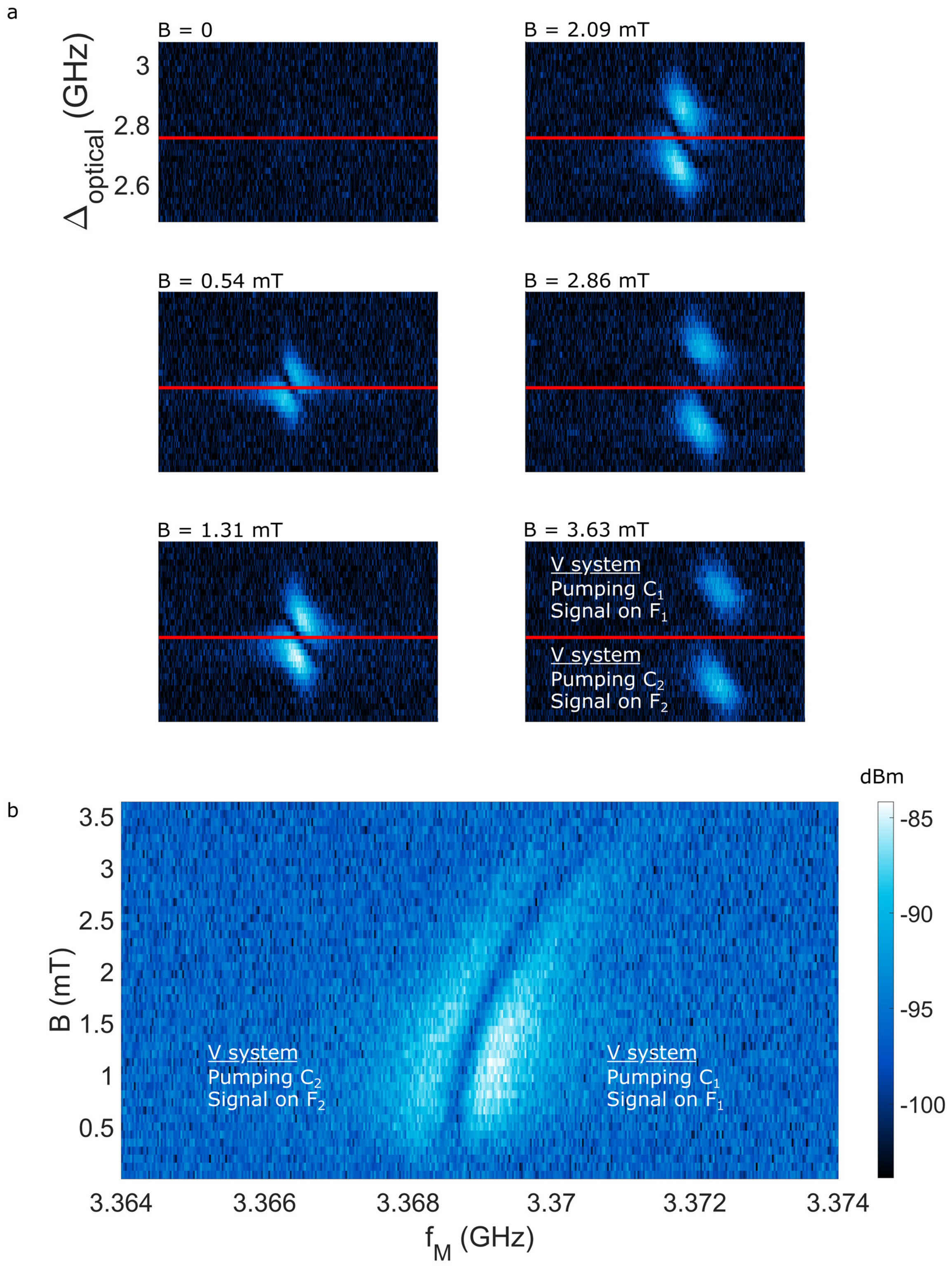

Supplementary Figure 8: Destructive interference of two frequency-degenerate V systems. a The transduction signal as a function of microwave input frequency f_M and optical pump frequency $\Delta_{\mathrm{optical}}$ at six magnetic field values. Note that the horizontal axis is the same as the plot in part (b). **b** Transduction signal as a function of microwave input frequency and applied magnetic field for a fixed laser frequency $\Delta_{\mathrm{optical}}$ = 2.75 GHz, indicated by the red lines in part (a).

The signals shown in Supplementary Figure 8 are generated by two V systems. The upper lobe corresponds to signals generated from the $|1\rangle_g \overset{C_1}{\leftrightarrow} |1\rangle_e \leftrightarrow |2\rangle_e \overset{F_1}{\leftrightarrow} |1\rangle_g$ system and the lower lobe is generated from the $|2\rangle_g \overset{C_2}{\leftrightarrow} |1\rangle_e \leftrightarrow |2\rangle_e \overset{F_2}{\leftrightarrow} |2\rangle_g$ system. At zero field, C_1 and C_2 are degenerate (transition C) as are F_1 and F_2 (transition F). The optical fields generated at the frequency of transition F possess the opposite phase and hence, destructively interfere. The difference in output field phase is due to the product of optical transition dipole moments involved in the transduction. Letting the electron spin be denoted as $|\uparrow\rangle$ or $|\downarrow\rangle$ and the nuclear spin denoted as $|\Uparrow\rangle$ or $|\Downarrow\rangle$, and factoring out common elements, the relative phase can be calculated from

$$\langle 1_g|S_x|1_e\rangle\langle 2_e|S_x|1_g\rangle = \langle\uparrow\Uparrow|S_x|\tfrac{1}{\sqrt{2}}(|\uparrow\Downarrow\rangle - |\downarrow\Uparrow\rangle)\,\tfrac{1}{\sqrt{2}}(\langle\uparrow\Downarrow| + \langle\downarrow\Uparrow|)|S_x|\uparrow\Uparrow\rangle \propto -1, \quad \text{and} \tag{6}$$

$$\langle 2_g|S_x|1_e\rangle\langle 2_e|S_x|2_g\rangle = \langle\downarrow\Downarrow|S_x|\tfrac{1}{\sqrt{2}}(|\uparrow\Downarrow\rangle - |\downarrow\Uparrow\rangle)\,\tfrac{1}{\sqrt{2}}(\langle\uparrow\Downarrow| + \langle\downarrow\Uparrow|)|S_x|\downarrow\Downarrow\rangle \propto 1, \tag{7}$$

which gives a π phase shift between the two output fields on F_1 and F_2 . (Note S_x is the electron spin operator).

It is clear from the detailed scans in Supplementary Figure 8.a that the spin transition frequency and optical transition frequency are correlated. This correlation of -120 MHz/MHz (Δ_{optical} / f_M) shows that there is structure underlying the inhomogeneous broadening on both spin and optical transitions. Correlations between optical and spin transition frequencies have been observed in other rare-earth ion systems, including Eu^{3+}:$YAlO_3$[15,16] and $EuCl_3.6H_2O$[17]. In these cases, which examined systems in which the electron-spin is quenched, the correlation was positive and of the order of 100 MHz/kHz (optical / spin). Modelling the impact of crystal field perturbations on the hyperfine levels would be an interesting extension of this work to determine whether the correlation is related to strain.

For this work, the correlation between optical and spin transition frequencies combined with the destructive interference to give rise to an interesting feature highlighted in Supplementary Figure 8.b. For B < 0.25 mT the two transitions interfere destructively and no transduction signal is observed. For 0.25 mT < B < 3.25 mT the two V-systems are partially overlapped resulting in two distinct microwave resonances. This is despite both signals arising from the common $|1\rangle_e \leftrightarrow |2\rangle_e$ transition. Furthermore, using phase sensitive detection, it is possible to observe the phase shift on the output optical signal as the microwave frequency is scanned across the spin transition (see Supplementary Figure 9). For B > 3.25 mT the two V-systems are completely resolved.

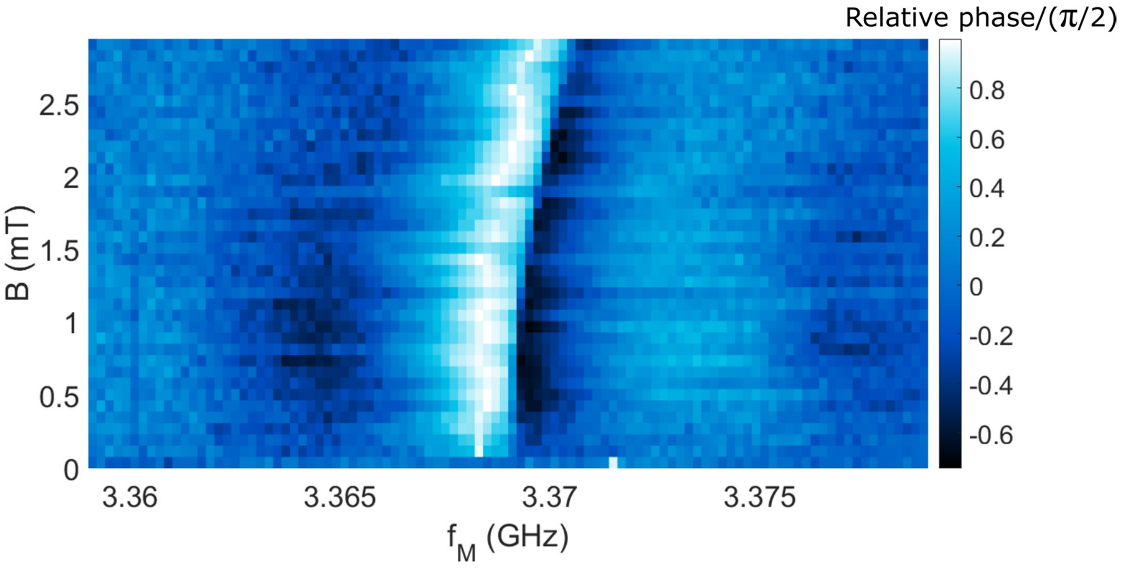

Supplementary Figure 9: Phase sensitive detection of the magnitude spectra shown in Supplementary Figure 8.b. The plot shows the opposite phases of the two transduction signals arising from the optical-spin frequency correlation and the π phase shift in the optical output field.

Supplementary Note 9 - Temperature

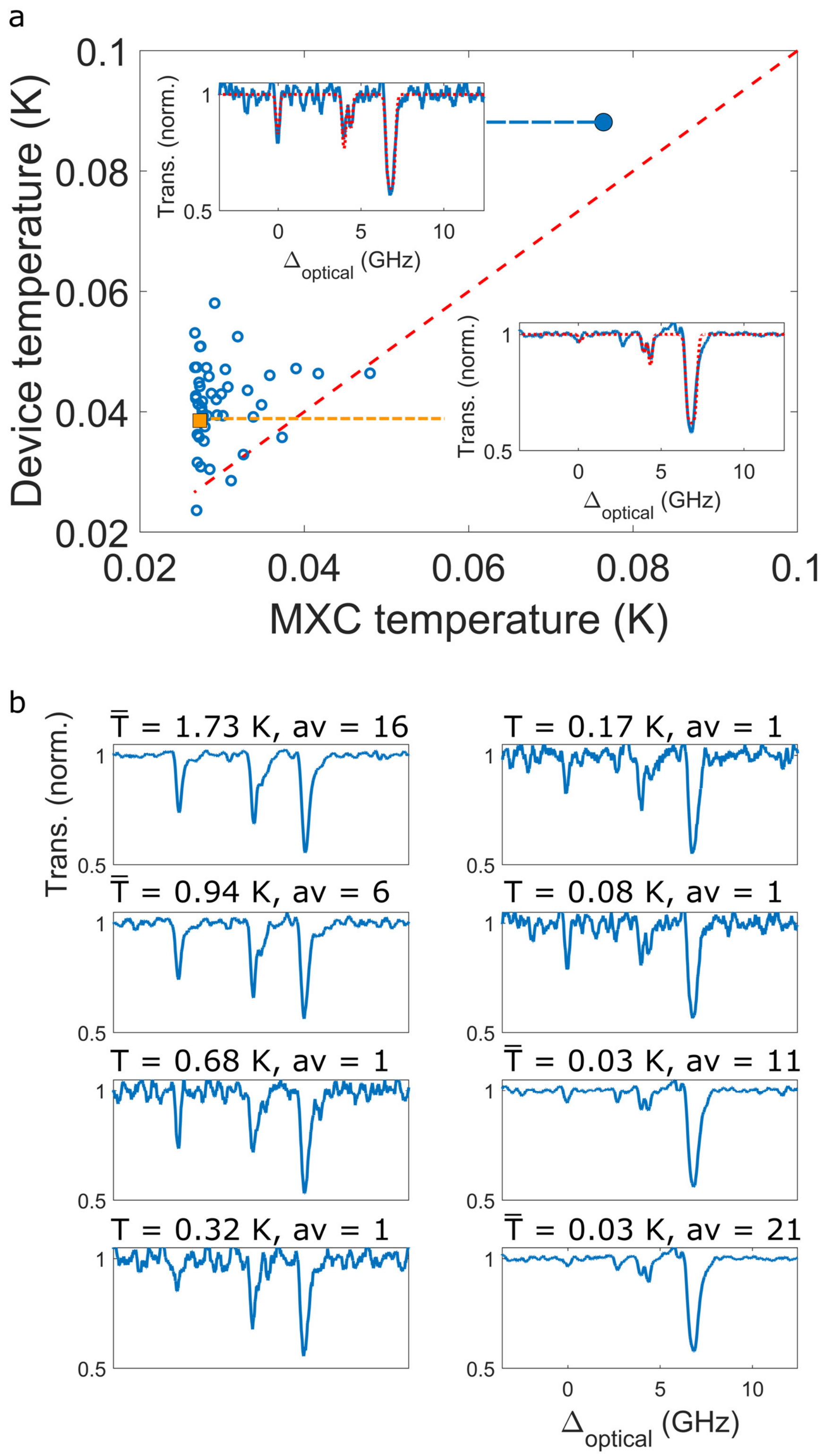

Supplementary Figure 10: Temperature of the $^{171}Yb^{3+}$-ion ensemble in the waveguide. a Device temperature data as a function of the mixing chamber (MXC) temperature during the refrigerator condensation cycle. The plotted data are measured from fitting transmission measurements (see insets for examples) at low probe power. The orange square represents the average of 21 measurements taken as the MXC approached its base temperature. **b** Further examples of the transmission measurements at different MXC temperatures *T* (mean temperatures $\overline{T}$ averaged over *av* samples) during the condensation cycle.

An important question for rare-earth ion quantum devices and protocols is whether the device – typically based on an insulating crystal – is attaining the base temperature of the cryostat. Two challenges to cool devices effectively are rapidly decreasing thermal conductivities at cryogenic temperatures, and the nanoscale cross sectional area for our suspended on-chip devices. We investigated the temperature of the on-chip waveguide by measuring the relative spin populations during the condensation cycle of the dilution refrigerator. During this cycle, the MXC temperature changes from ≈4 K to a base temperature of ≈30 mK over a period of approximately 2 hours.

The measurement consisted of recording transmission spectra of the $^{171}Yb^{3+}$ ions with low excitation powers during the cool down. The results are shown in Supplementary Figure 10. When the temperature was changing slowly it was possible to average over several spectra for more accurate data but when the temperature changed rapidly we rely on a single spectrum. Each transmission spectrum is fitted to a spin Hamiltonian prediction that included both the ^{171}Yb and ^{even}Yb isotopes. Given GDMS measurements of the relative abundance of each isotope in the sample, the model accounted for detected light that does not pass through the waveguide. The fitting process yields the temperature of the $^{171}Yb^{3+}$ ions, which reach a minimum of 40 ± 10 mK as shown in Supplementary Figure 10.a. Supplementary Figure 10.b shows further spectra at different MXC temperatures.

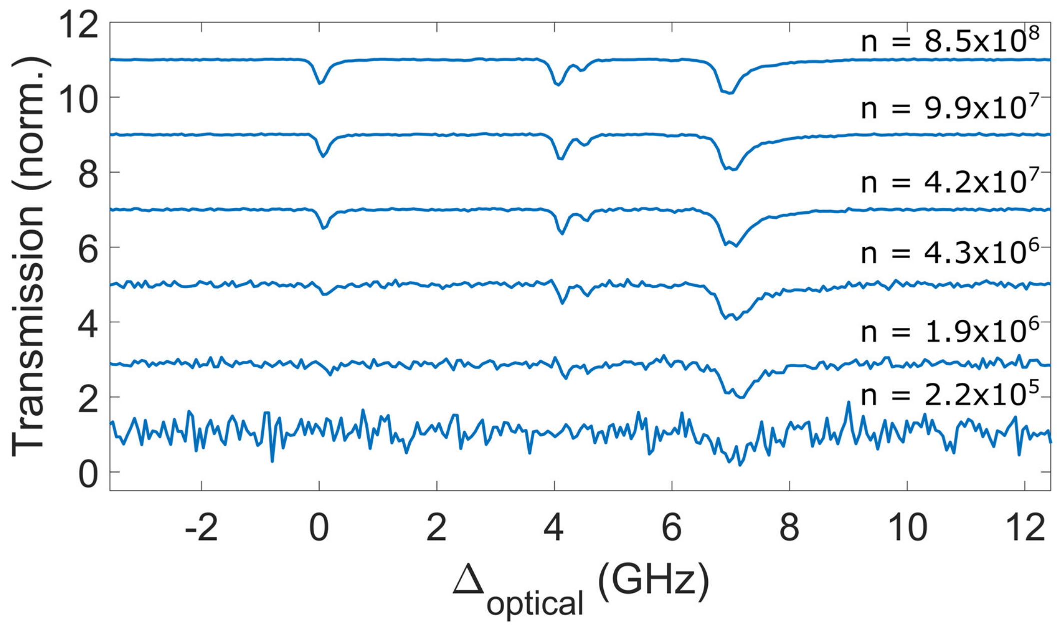

Supplementary Figure 11: Transmission measurements of the $^{171}Yb^{3+}$-doped waveguide as a function of probe intensity. The intensity n is the number of photons per second incident on the chip.

Supplementary Figure 11 shows further data taken with the MXC at base temperature, where we probe the ensemble temperature as a function of probe intensity. In Supplementary Figure 11, n refers to the number of photons incident on the YVO chip per second, about 20% of which couple to the waveguide. The results show that the ensemble temperature increases to >100 mK with 10^7 photons per second on-chip. The device temperature during CW transduction measurements was estimated to be approximately 1 K by monitoring the transduction efficiency as a function of MXC temperature.

Supplementary Note 10 - All optical measurements of spin state lifetime and optical coherence

The ground state spin lifetime T_1 and the optical coherence lifetime $T_{2\,(\mathrm{Optical})}$ are two further parameters of interest that are relevant to the ultimate performance limits of the transducer and the broader quantum technology applications of this material. These measurements used LS_2 (Supplementary Figure 1.d) for excitation and the gated APD path for detection. Supplementary Figure 12.a shows the pulse sequences used for both measurements.

Measurements of the spin T_1 are important to assess the potential for optical initialization into a single ground state. The population in $|4\rangle_g$ was depleted using a series of optical π pulses separated by the optical lifetime (300 µs). A further π pulse was then used to read out the repopulation of $|4\rangle_g$ as a function of the wait time. The population recovery was found to be composed of two timescales. The shorter timescale (T_1 = 12.5 ms) is shown in Supplementary Figure 12.b and is likely to correspond to the $|3\rangle_g \rightarrow |4\rangle_g$ transition. The longer timescale was found to be ≫40 ms, which is attributed to the $|1\rangle_g, |2\rangle_g \rightarrow |4\rangle_g$ transition.

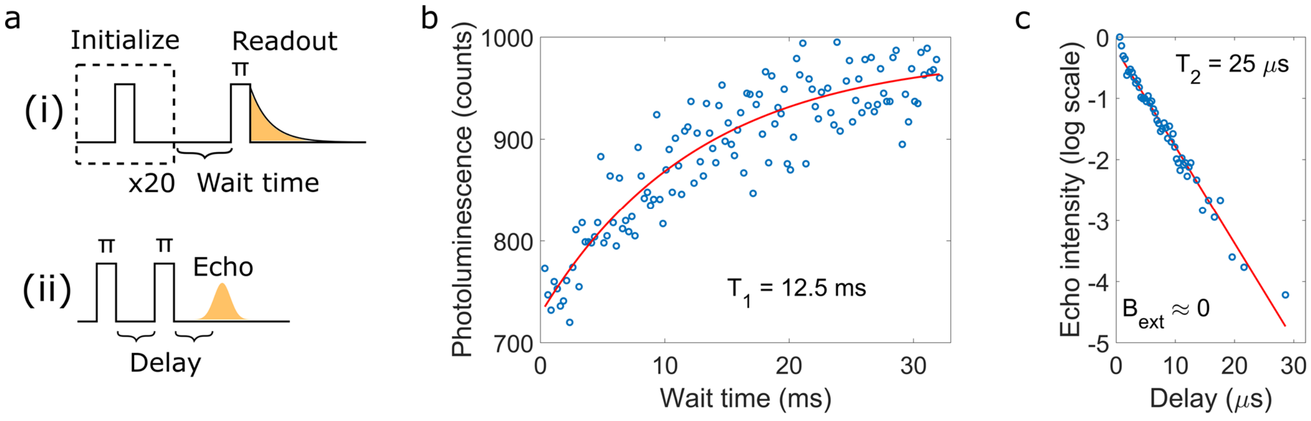

Supplementary Figure 12: Optical measurements of spin state lifetime and optical coherence lifetime. a Pulse sequence used for the two measurements: (i) - part (b) and (ii) – part (c). **b** Relaxation lifetime T_1 of population from $|3\rangle_g \rightarrow |4\rangle_g$ measured by population recovery. **c** Optical echo intensity as a function of delay time for the A transition ($\Delta_{\mathrm{optical}}$ = 0) at zero field.

The optical coherence lifetime was measured for transition A: one of the optical clock transitions[18,19]. The optical pulse sequence is shown in Supplementary Figure 12.a(ii) and the results are plotted in Supplementary Figure 12.c . $T_{2\,(\mathrm{Optical})}$ was found to be 25 µs and is likely to be dominated by Yb-Yb interactions given the high spectral density of $^{171}Yb^{3+}$ in this material.

Further studies are necessary to gain a more complete picture of the contributing mechanisms to these relaxation rates.

Supplementary References

1. E. C. Cook, P. J. Martin, T. L. Brown-Heft, J. C. Garman & D. A. Steck, High passive-stability diode-laser design for use in atomic-physics experiments. *Rev. Sci. Instrum* **83**, 43101 (2012).

2. T. Zhong, J. Rochman, J. M. Kindem, E. Miyazono & A. Faraon, High quality factor nanophotonic resonators in bulk rare-earth doped crystals. *Opt. Express* **24**, 536 (2016).

3. L. A. Williamson, Y.-H. Chen & J. J. Longdell, Magneto-optic modulator with unit quantum efficiency. *Phys. Rev. Lett.* **113**, 1–5 (2014).

4. Fernandez-Gonzalvo, X. Coherent Frequency Conversion from Microwave to Optical Fields in an Erbium Doped Y2SiO5 Crystal: Towards the Single Photon Regime. PhD thesis, University of Otago (2017).

5. G. Dold *et al.*, High-Cooperativity Coupling of a Rare-Earth Spin Ensemble to a Superconducting Resonator Using Yttrium Orthosilicate as a Substrate. *Phys. Rev. Appl.* **10**, 54082 (2019).

6. M. V. Jacob, J. Mazierska & J. Krupka, Dielectric Properties of Yttrium Vanadate Crystals from 15 K to 295 K. *J. Electroceramics* **15**, 237–241 (2005).

7. X. Fernandez-Gonzalvo, Y.-H. Chen, C. Yin, S. Rogge & J. J. Longdell, Coherent frequency up-conversion of microwaves to the optical telecommunications band in an Er:YSO crystal. *Phys. Rev. A* **92**, 062313 (2015).

8. X. Fernandez-Gonzalvo, S. P. Horvath, Y.-H. Chen & J. J. Longdell, Cavity-enhanced Raman heterodyne spectroscopy in Er^{3+}:Y_2SiO_5. *Phys. Rev. A* **100**, 033807 (2019).

9. F. J. P. Schuurmans, P. de Vries & A. Lagendijk, Local-field effects on spontaneous emission of impurity atoms in homogeneous dielectrics. *Phys. Lett. A* **264**, 472–477 (2000).

10. H. T. Dung, S. Y. Buhmann & D.-G. Welsch, Local-field correction to the spontaneous decay rate of atoms embedded in bodies of finite size. *Phys. Rev. A* **74**, 023803 (2006).

11. J. M. Kindem *et al.*, Characterization of $^{171}Yb^{3+}$:YVO_4 for photonic quantum technologies. *Phys. Rev. B* **98**, 024404 (2018).

12. Kindem, J. M. *et al.* Control and single-shot readout of an ion embedded in a nanophotonic cavity. *Nature* **580**, 201–204 (2020).

13. T. G. Tiecke *et al.*, Efficient fiber-optical interface for nanophotonic devices. *Optica* **2**, 70 (2015).

14. J. P. Covey, A. Sipahigil & M. Saffman, Microwave-to-optical conversion via four-wave mixing in a cold ytterbium ensemble. *Phys. Rev. A* **100**, 012307 (2019).

15. L. E. Erickson & K. K. Sharma, Nuclear quadrupole resonance measurements of the anisotropic magnetic shielding and quadrupole coupling constants of $^{151}Eu^{3+}$ and $^{153}Eu^{3+}$ dilute in $YAlO_3$ single crystal. *Phys. Rev. B* **24**, 3697–3700 (1981).

16. M. Yamaguchi, K. Koyama, T. Suemoto & M. Mitsunaga, Mapping of site distribution in Eu^{3+}: $YAlO_3$ on RF-optical frequency axes by using double-resonance spectroscopy. *J. Lumin.* **76**, 681–684 (1998).

17. R. L. Ahlefeldt, A. Smith & M. J. Sellars, Ligand isotope structure of the optical $^7F_0$ - $^5D_0$ transition in $EuCl_3.6H_2O$. *Phys. Rev. B* **80**, 5–9 (2009).

18. A. Tiranov *et al.*, Spectroscopic study of hyperfine properties in $^{171}Yb^{3+}$:Y_2SiO_5. *Phys. Rev. B* **98**, 195110 (2018).

19. A. Ortu *et al.*, Simultaneous coherence enhancement of optical and microwave transitions in solid-state electronic spins. *Nat. Mater.* **17**, 671–675 (2018).
